# A new leiognathid record from China with complete mitogenomes and phylogenetic insights of two *Aurigequula* (Teleostei, Leiognathidae) species

**DOI:** 10.3897/zookeys.1267.174380

**Published:** 2026-01-21

**Authors:** Jia-Jie Chen, Jun-Sheng Zhong, Sheng Zeng, De-Yuan Yang, Pan Liu, Xiao-Dong Wang, Han-Ye Zhang, Jin-Qing Ye

**Affiliations:** 1 Shanghai Universities Key Laboratory of Marine Animal Taxonomy and Evolution, Shanghai Ocean University, 201306, Shanghai, China Xiamen University Xiamen China; 2 East China Sea Fisheries Research Institute, Fisheries Science of Chinese Academy, 200090, Shanghai, China Shanghai Natural History Museum, Branch of Shanghai Science and Technology Museum Shanghai China; 3 State Key Laboratory of Marine Environmental Science, College of Ocean and Earth Sciences, Xiamen University, Xiamen 361102, China National Marine Environment Monitoring Center Dalian China; 4 Conversation and Research Center for Collections, Shanghai Natural History Museum, Branch of Shanghai Science and Technology Museum, 200041, Shanghai, China Shanghai Ocean University Shanghai China; 5 National Marine Environment Monitoring Center, 116023, Dalian, China East China Sea Fisheries Research Institute, Fisheries Science of Chinese Academy Shanghai China

**Keywords:** *Aurigequula striata*, Hainan Island, Leiognathidae, mitochondrial genome, phylogenetic analysis, South China sea

## Abstract

The ponyfish genus *Aurigequula* is a relatively poorly studied group within the Leiognathidae, with its diversity and phylogenetic relationships in the West Pacific remaining unclear. This study reports the first confirmed record of *Aurigequula striata* from Chinese waters, based on two specimens obtained from a fish market in Sanya, Hainan Island. Newly collected specimens of *A. fasciata* from the same region are also provided, which enabled detailed morphological and genomic analysis. The morphological descriptions are supplemented by detailed photographic documentation of fresh and preserved specimens, addressing the historical scarcity of reliable visual references for these species. Furthermore, we present the first complete mitochondrial genomes for both *A. striata* (16,629 bp) and *A. fasciata* (16,537 bp). Our phylogenomic analysis, based on 13 protein-coding genes, strongly supports the monophyly of the subfamilies Gazzinae and Leiognathinae. Within Leiognathinae, however, the analysis suggests paraphyly of *Aurigequula*, with *A. fasciata* forming a clade with *Leiognathus equula* to the exclusion of *A. striata*. This result conflicts with the current morphology-based generic diagnosis, which relies on consistent differences in the length of the second dorsal-fin spine and body coloration patterns. This study significantly expands the genomic resources for the understudied genus *Aurigequula* but also highlights the limitations of relying solely on mitochondrial data for resolving generic-level relationships. A more robust phylogenetic framework for Leiognathidae, capable of reconciling molecular and morphological evidence, will require the integration of multiple independent nuclear loci.

## Introduction

The family Leiognathidae (ponyfishes), comprising small to medium-sized fishes inhabiting coastal marine and brackish waters of the Indo-West Pacific, is recognized for its taxonomic complexity and morphological conservatism (Chakrabarty and Sparks 2008; Chakrabarty et al. 2008). Recent systematic revisions, integrating molecular data with morphological re-examinations, have significantly advanced our understanding of their phylogeny and classification (Ikejima et al. 2004; Chakrabarty et al. 2011; Seah et al. 2012; Sparks and Chakrabarty 2015; Chen et al. 2024). Despite these efforts, the generic boundaries and species diversity within certain lineages, particularly the genus *Aurigequula*, remain inadequately resolved.

The genus *Aurigequula* was resurrected by Chakrabarty et al. (2008) and is currently considered to contain three valid species: the widely distributed Indo-West Pacific *A. fasciata* (Lacepède, 1803), *A. striata* (James & Badrudeen, 1991) – previously known only from the Indian Ocean (Gulf of Mannar, Sri Lanka) and Singapore (James and Badrudeen 1990; Chakrabarty and Sparks 2008, 2022), and *A. longispinis* (Valenciennes, 1835), also distributed in the Indo-West Pacific (Chakrabarty and Sparks 2022). In Chinese waters, only *A. fasciata* has been sporadically recorded (Zheng 1962; Chakrabarty et al. 2010). While *A. longispinis* has been listed in regional records (Sun and Chen 2013; Chen and Ni 2021), no verified specimens have been documented, and reliable records of *A. striata* were previously lacking.

The mitochondrial genome, with its maternal inheritance, lack of recombination, and relatively high evolutionary rate, has become a commonly used marker for inferring preliminary phylogenetic relationships and studying population genetics due to its maternal inheritance, lack of recombination, and relatively high evolutionary rate (Zhang and Hewitt 1997; Satoh et al. 2016). Its sequence data can provide a useful preliminary framework for phylogenetic studies, which can then be tested and refined with data from independent nuclear loci. Complete mitogenomes have been increasingly used to elucidate the systematics of various fish groups, including leiognathids (Shi et al. 2018; Sui et al. 2019; Chen et al. 2024). However, no complete mitogenome has been available for any *Aurigequula* species, limiting a comprehensive understanding of their phylogenetic placement.

In this study, we document the first record of *A. striata* from China, providing detailed morphological descriptions and illustrations; report new specimens of *A. fasciata* from Hainan Island; present and characterize the complete mitochondrial genomes of both species; and reconstruct a mitogenome-based phylogeny of Leiognathidae to assess the phylogenetic position and relationships of the two *Aurigequula* species.

## Materials and methods

### Sample collection, morphology, and DNA extraction

**Figure 1.**
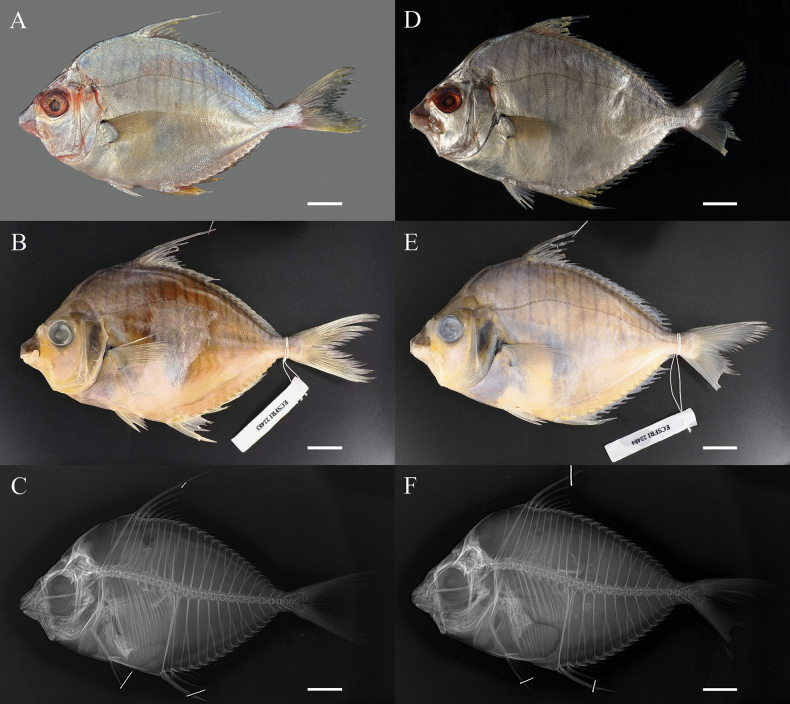
*Aurigequula striata*, left lateral view. **A–C**. ECSFRI 22483, 156.75 mm SL; **D–F**. ECSFRI 22484, 152.62 mm SL. **A, D**. Fresh coloration after thawing; **B, E**. Preserved specimen; **C, F**. Radiograph. Shaded area indicates muscle tissue sampling location below dorsal fin. Photos by JC. Scale bar: 20 mm.

**Figure 2.**
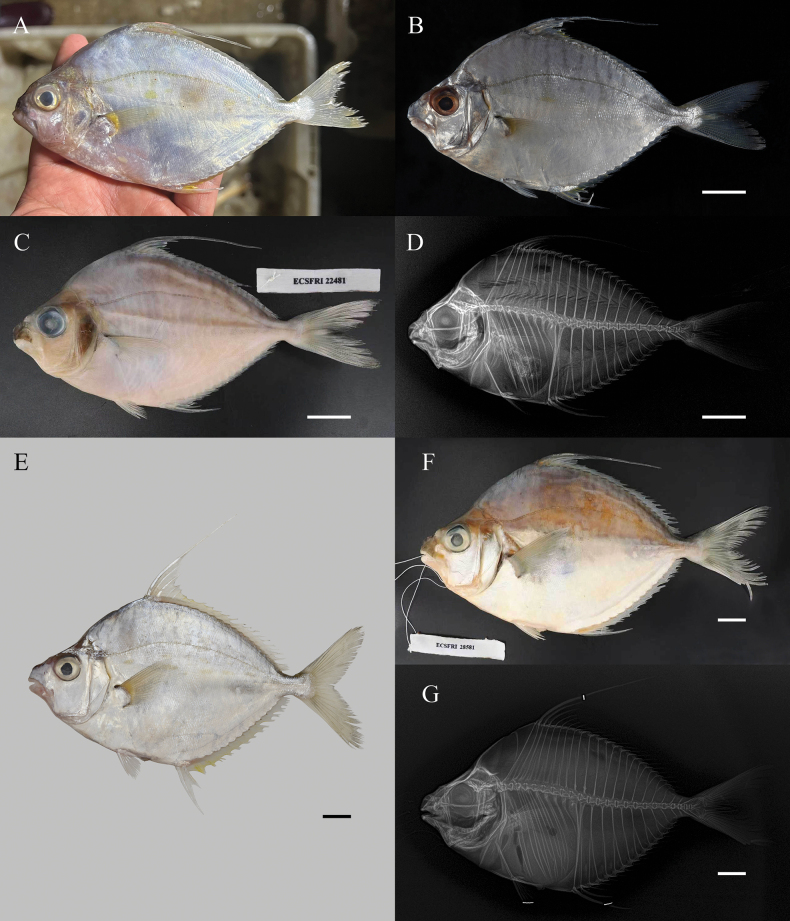
*Aurigequula fasciata*, left lateral view. **A–D**. ECSFRI 22481, 116.95 mm SL; **E–G**. ECSFRI 22581, 188.01 mm SL. **A** Freshly landed specimen at dockside; **B, E**. Fresh coloration after thawing (**B**) and live coloration (**E**); **C, F**. Preserved specimens; **D, G**. Radiographs. Shaded area indicates muscle tissue sampling location below dorsal fin. Photos by JC (**A–D, F, G**) and PL (**E**). Scale bar: 20 mm.

All specimens are deposited at the East China Sea Fisheries Research Institute (**ECSFRI**), Shanghai, China. The material examined includes two specimens of *A. striata* (voucher nos. ECSFRI 22483–22484) were collected at Hongsha Fish Market in Sanya, Hainan Island, on 7 November 2023 (Fig. 1A–C). One specimen of *A. fasciata* (ECSFRI 22481) was obtained from Xincun Port in Lingshui, Hainan Island (Fig. 1E, F). Two additional specimens of *A. fasciata* were also included in the study: ECSFRI 22488 (Fig. 2A–D), collected at Hongsha Fish Market on 7 November 2023, and ECSFRI 28581 (Fig. 2E–G), obtained from a fish market in Sanya in late April 2025. To enhance phylogenetic resolution, three specimens of *Leiognathus equula* were added: ECSFRI 22489 from Hongsha Fish Market on 7 November 2023, ECSFRI 25000 from Qinglan, Hainan in November 2023, and ECSFRI 19056 from Yangxi, Guangdong on 26 October 2023 (Suppl. material 1: fig. S1). All specimens were fixed in 10% formalin and subsequently preserved in 70% ethanol.

Species identification was based on morphological characters following Zheng (1962), James and Badrudeen (1990), Chakrabarty et al. (2008), Chakrabarty and Sparks (2022), Chen and Ni (2021). Methods for morphometric analysis followed Sparks and Dunlap (2004), Hubbs and Lagler (2004) and Chen et al. (2024), with the hypural plate counted as a single vertebra. Prior to DNA isolation, the specimen surface was cleaned with 100% ethanol. Approximately 10 × 10 mm^2^ of muscle tissue was excised from below the right dorsal fin for genomic DNA extraction. Total genomic DNA was extracted using the TIANamp Genomic DNA Kit (TIANGEN, Beijing, China). All specimens were initially fixed in 10% formalin and subsequently transferred to 70% ethanol for long-term preservation.

### Mitogenome sequencing and assembly

The mitochondrial genomes were sequenced and assembled following established protocols (Chen et al. 2024; Xu et al. 2024; Yang et al. 2024). DNA libraries were prepared with the Illumina TruSeqTM DNA Sample Preparation Kit (Illumina, USA) following manufacturer guidelines. Novogene Bioinformatics Technology Co., Ltd. (China) conducted sequencing on a DNBSEQ-T7 platform, generating 150 bp paired-end reads yielding ~5 Gb raw data per sample. Data preprocessing involved Fastp v. 0.23.2 (Chen et al. 2018) with default parameters and quality assessment using FastQC v0.12.1 (http://www.bioinformatics.babraham.ac.uk/projects/fastqc/). Mitogenome assembly employed the FastMitoAssembler pipeline, augmented by GetOrganelle v. 1.7.6.1 (Jin et al. 2020) and NovoPlasty v. 4.3.1 (Dierckxsens et al. 2017).

### Mitogenome annotation and sequence analyses

The complete mitochondrial genome was annotated using MITOS2 (Donath et al. 2019) and Mitoz v. 3.6 (Meng et al. 2019), with manual validation in Geneious v2021.0.3. PhyloSuite v. 1.2.3 (Zhang et al. 2020) was employed to quantify base composition, codon usage, and relative synonymous codon usage (RSCU) of protein-coding genes (PCGs). Nucleotide skewness (A+T skew = [A%−T%]/[A%+T%]; G+C skew = [G%−C%]/[G%+C%]) was calculated following Perna and Kocher (1995). Selective pressure analysis through Ka/Ks ratios was conducted for Leiognathidae species mitogenomes using DnaSP 6.0 (Rozas et al. 2017), and results were visualized using Origin 8. Transfer RNA secondary structures were predicted using MITOS2 (Arab et al. 2017; Donath et al. 2019) and visualized using Python-based bioinformatics packages.

Based on the identical mitogenomes observed among the two *A. striata* individuals and the three *A. fasciata* individuals, a single representative specimen for each species—voucher number ECSFRI 22483 (Accession no. PX227131) for *A. striata* and ECSFRI 22485 (Accession no. PX227128) for *A. fasciata*—was selected for detailed description of the complete mitochondrial genome.

### Phylogenetic analysis

We reconstructed the Leiognadidae phylogeny using a total of 16 mitogenomic sequences, comprising 10 species, with *Naso hexacanthus* and *Chaetodon modestus* designated as outgroups. Mitogenome sequences retrieved from GenBank (Suppl. material 1: table SS1) were processed in PhyloSuite 1.2.3 (Zhang et al. 2020). Thirteen protein-coding genes (PCGs) were aligned codon-wise using MAFFT v. 7.313 (Katoh and Standley 2013). ModelFinder v. 2.2.0 (Kalyaanamoorthy et al. 2017) was used to select the best substitution models under the Bayesian Information Criterion (BIC) for maximum likelihood (ML) analysis and the corrected Akaike Information Criterion (AICc) for Bayesian inference (BI) analysis (Suppl. material 1: table S2).

Maximum likelihood analysis employed IQ-TREE v. 2.2.2 with edge-linked partitioning (100,000 ultrafast bootstrap replicates), while Bayesian inference utilized MrBayes v. 3.2.7a (Ronquist et al. 2012; two parallel runs; 2 × 10^6^ generations). Final topologies were visualized using iTOL v. 6 (Letunic and Bork 2016).

## Results

### Species descriptions

#### Aurigequula striata

Taxon classificationAnimaliaAcanthuriformesLeiognathidae

(James & Badrudeen, 1990)

5BE557B5-57D3-5708-8CBD-BE974225642F

##### Diagnosis.

D VIII 16; A III 14; P I 19–20; V I-5; Vertebrae 24. Body robust and thick. Flank with 12–15 distinct vertical bars. Second dorsal fin spine elongated as filament.

##### Description.

Based on 2 specimens, 152.62–156.75 mm SL. Proportional measurements and counts are given in Table 1.

**Table 1.** Comparison of morphometric and meristic characters of *A. striata* and *A. fasciata* from the Western Pacific and the Indian Ocean.

Counts and measurements	Western Pacific (this study)		Indian Ocean (Chakrabarty et al. 2008)	
	*A. striata* (*n* = 2)	*A. fasciata* (*n* = 3)	*A. striata* (*n* = 3)	*A. fasciata* (*n* = 3)
Standard length (mm)	154.69 (152.62–156.75)	130.40 (86.23–188.01)	89.5–173.6	112.3–124.6
Counts				
Dorsal fin rays	VIII 16	VIII 16	–	–
Anal fin rays	III 14	III 14	–	–
Pectoral fin rays	I 19–20	I 17–19	–	–
Pelvic fin rays	I 5	I 5	–	–
Lateral line scales	67 (64–69)	60 (55–64)	–	–
Scale rows above lateral line	21 (20–21)	22 (21–22)	–	–
Scale rows below lateral line	46 (43–48)	44 (41–47)	–	–
Vertebrae	24	24	–	–
Gill rakers on upper arch	5 (4–6)	3 (3–4)	–	–
Gill rakers on lower arch	18 (17–18)	14 (13–14)	–	–
Total gill rakers	23 (21–24)	17 (17–17)	–	–
Measurements				
As % of standard length				
Head length	29.71 (29.47–29.94)	30.54 (29.57–31.39)	33.2 (31.9–35.1)	32.0 (30.9–33.9)
Head depth (max.)	47.47 (47.42–47.52)	46.30 (44.99–47.99)	–	–
Body depth	54.64 (53.89–55.38)	54.39 (52.22–57.21)	59.5 (57.9–60.7)	54.8 (53.2–57.1)
Caudal peduncle length	8.47 (8.16–8.79)	8.79 (8.44–9.29)	8.3 (7.0–9.3)	8.0 (7.7–8.4)
Caudal peduncle depth	7.43 (7.32–7.53)	6.78 (6.68–6.84)	6.8 (6.1–7.3)	6.7 (6.4–7.1)
Caudal peduncle width	3.93 (3.78–4.08)	3.50 (2.80–4.18)	4.3 (4.0–4.8)	4.6 (4.5–4.7)
Pectoral-fin length	23.45 (23.22–23.69)	22.42 (22.04–22.67)	20.5 (18.7–21.5)	22.7 (22.5–22.9)
Pelvic-fin length	15.18 (14.98–15.37)	13.86 (13.45–14.08)	15.0 (12.3–18.1)	13.2 (12.9–13.4)
Dorsal fin base length	57.66 (57.40–57.91)	55.99 (55.33–56.81)	–	–
Pectoral fin base length	8.12 (7.95–8.29)	7.33 (7.04–7.87)	–	–
Pelvic fin base length	4.38 (4.05–4.70)	3.26 (2.10–4.28)	–	–
Anal fin base length	47.27 (46.94–47.60)	45.64 (44.69–46.26)	–	–
Predorsal length	34.37 (34.09–34.65)	39.09 (37.91–39.80)	54.1 (52.8–55.2)	52.1 (51.3–53.5)
Prepelvic length	31.95 (31.32–32.57)	34.41 (32.38–35.82)	39.6 (37.4–40.7)	42.0 (40.5–42.9)
Preanal length	49.65 (49.18–50.12)	52.18 (50.58–53.39)	59.2 (54.9–66.5)	57.7 (56.5–58.4)
Prepectoral length	31.05 (30.83–31.27)	31.93 (31.64–32.22)	–	–
As % of head length				
Snout length	32.29 (31.06–33.53)	30.27 (29.81–30.66)	37.4 (33.0–41.7)	32.9 (32.0–34.0)
Head width (max.)	52.13 (50.25–54.00)	47.00 (45.97–48.58)	–	–
Upper jaw length	40.70 (40.62–40.78)	37.83 (35.74–39.90)	38.7 (34.6–42.4)	40.5 (38.4–43.4)
Lower jaw length	45.97 (44.39–47.55)	44.40 (43.81–44.77)	34.6 (25.8–50.3)	49.3 (45.0–51.6)
Interorbital width	33.00 (31.15–34.84)	33.85 (33.32–34.36)	31.3 (25.8–35.2)	34.0 (28.0–37.1)
Eye diameter/Orbit diameter	29.22 (28.33–30.12)	32.22 (30.80–33.58)	31.1 (28.6–33.9)	34.5 (32.9–35.5)
Preorbital depth	71.41 (68.92–73.90)	68.93 (67.17–70.37)	–	–
First dorsal fin spine length	7.30 (6.87–7.73)	6.64 (4.58–7.72)	–	–
Second dorsal fin spine length	87.94 (69.99*–105.89)	147.75 (140.59–154.92)	–	–
Third dorsal fin spine length	61.00 (55.13–66.86)	46.85 (38.75–54.95)	–	–
First anal fin spine length	11.18 (11.00–11.36)	7.44 (6.30–8.27)	–	–
Second anal fin spine length	69.85 (66.62–73.08)	67.40 (66.89–67.92)	–	–
Third anal fin spine length	45.97 (44.78–47.17)	39.85 (37.16–42.74)	–	–
Pectoral fin spine length	24.33 (21.67–26.99)	20.58 (18.91–21.50)	–	–
Pelvic fin spine length	46.18 (45.89–46.47)	36.88 (34.20–38.79)	–	–

Notes: An asterisk (*) denotes a damaged specimen. A dash (–) indicates that the corresponding data were not provided in the original source.

Body large, robust, laterally compressed; deep-bodied (53.89–55.38% SL). Dorsal profile more convex than ventral; greatest depth below dorsal-fin origin. Dorsal- and pelvic-fin origins on same vertical axis; dorsal origin posterior to pelvic base. Anal-fin origin below first–second dorsal-fin ray interval (Fig. 1C, F). Dorsum of head with flattened triangular area enclosed by two supraorbital ridges; posterior ends of ridges connecting to nuchal spine base, slightly depressed. Dorsal profile of head moderately convex; nuchal spine longer than eye diameter (Fig. 1). Snout truncated; length 31.06–33.53% HL, greater than eye diameter (28.33–30.12% HL); tip blunt, squared. Gill opening large. Lower preopercular margin right-angled, weakly serrate. Branchiostegal rays 5; membrane attached along lateral isthmus margin. Caudal peduncle short (8.16–8.79% SL), shallow (7.32–7.53% SL). Vertebrae 24. Neural and haemal spines of PU4 expanded, bladelike (Fig. 1C, F).

Dorsal fin I, VIII,16; spine 1 shortest; spine 2 longest (105.89% HL, ECSFRI 22483); spines 3–4 anteriorly serrate; anal fin III,14; spine 2 longest (66.62–73.08% HL); spines anteriorly serrate; dorsal and anal fin bases with membranous sheath anteriorly; pectoral fin rounded, broad; pelvic fin subthoracic, shorter than pectoral; large axillary scale present; spines retractile; caudal fin forked; lobes rounded, blunt-tipped.

Mouth small, terminal, oblique; tubular when protruded; cleft below lower orbital margin; lower jaw strongly concave; lips fleshy, thin; maxilla exposed, extending beyond anterior orbital margin. Teeth small, villiform, pointed and bristled, with incurved tips; arranged in three or four rows forming bands on both upper and lower jaws; vomer, palatine, and tongue edentulous.

Eye moderately large, high-set, its lower margin above body axis; preorbital spine ridged and serrated; adipose eyelid poorly developed; interorbital space slightly convex; short spine present on anterosuperior orbital margin, posterior to nostril; nostrils dorsal to eye, two on each side; anterior nasal pore small and round, posterior large and oblong.

Body covered with cycloid scales; head, breast, and large diagonal region of nuchal spine asquamate; lateral line complete, slightly arched from upper opercle to end of dorsal-fin base, then horizontal along caudal peduncle; pored scales 64–69; pelvic and anal fins each with large axillary scale.

Pigmentation. Body silvery; head and asquamate chest region silvery-white; dorsal flank with 12–15 gray vertical bars extending to lateral midline; opercle yellowish; snout with dense melanophores dorsally above upper lip; fin spines silvery; pectoral-fin base and axil unpigmented; interradial membranes of dorsal, anal and caudal fins tinged light yellow; pelvic fins white, with silvery-white axillary scale; anal fin silvery-white (Fig. 1A, D). In preservative: Body yellowish; snout blackish due to concentrated melanophores; vertical bars on dorsal flank darkened to black (Fig. 1B, E).

##### Distribution.

First recorded from the Gulf of Mannar (Pamban, Mandapam, and Kilakarai), India, in the Indian Ocean (James and Badrudeen 1990), with subsequent reports from Sri Lanka (Chakrabarty and Sparks 2008; Chakrabarty et al. 2008) and Singapore (Chakrabarty and Sparks 2022). The present study confirms its first reliable record in the West Pacific, based on specimens from Hainan Island, China.

##### Remarks.

Two specimens of *A. striata* were discovered incidentally among museum lots previously identified as *Leiognathus equula*, a common species in southern Chinese waters (Chen et al. 2024). *A. striata* appears to be rare and has only been recorded from a single locality (Hongsha Fish Market, Hainan). This species resembles *L. equula* in having faint dorsal stripes after color fading, but differs in possessing broader and more distinct stripes. In *L. equula*, the stripes are finer and generally only visible in less fresh specimens, remaining much less conspicuous than those of *A. striata*. Additionally, *A. striata* can be distinguished from *L. equula* by its longer second dorsal-fin spine (105.89% HL vs 80.35%, range 77.50–86.39% HL) and shorter head length (29.71% SL, range 29.47–29.94% SL vs 33.21% SL, range 31.47–35.91% SL) (Table 1; Chen et al. 2024).

#### Aurigequula fasciata

Taxon classificationAnimaliaAcanthuriformesLeiognathidae

(Lacepède, 1803)

913E8610-AE60-5057-BB01-5AB89DC2C979

##### Diagnosis.

D VIII 16; A III 14; P I 19–20; V I-5; Vertebrae 24. Body robust and thick. Flank with 12–14 distinct vertical bars, ventral flank with a series of rounded yellow dashes; three or four are enlarged and particularly distinct, forming the most conspicuous spots. Second dorsal fin spine elongated as filament.

##### Description.

Based on 3 specimens, 86.23–188.01 mm SL. Proportional measurements and counts are given in Table 1.

Body large, robust, laterally compressed, deep‐bodied (52.22–57.21% SL). Dorsal and ventral profiles equally convex; greatest depth at vertical between dorsal‐ and pelvic‐fin origins. Dorsal and pelvic origins vertically aligned (Fig. 2D, G). Head dorsum with flattened triangular area between supraorbital ridges; ridges extending to nuchal spine base, forming slight depression. Dorsal head profile moderately convex; nuchal spine shorter than eye diameter (Fig. 2). Snout truncated, length 29.81–30.66% HL, less than eye diameter (30.80–33.58% HL); tip blunt, squared. Gill opening large. Ventral preopercular margin right‐angled, weakly serrate. Branchiostegal rays 5; membrane attached along lateral isthmus. Caudal peduncle short (8.44–9.29% SL), shallow (6.68–6.84% SL). Vertebrae 24. Neural and haemal spines of PU4 expanded, bladelike (Fig. 2D, G). Dorsal fin I, VIII,16; spine 1 shortest; spine 2 longest (140.59–154.92% HL). Anal fin III,14; spine 2 longest (66.89–67.92% HL). Pored scales 55–64. Other characteristics as in *A. striata*.

Pigmentation. Body and head silvery-white, including asquamate breast region. Dorsal flank with 12–14 broad vertical bars extending just below lateral line, fragmenting into 3–4 rounded yellow dashes ventrally; bars yellow in fresh specimen (Fig. 2A), grayish in faded ones (Fig. 2B, E). All fin membranes faintly yellowish, most pronounced in anal-fin interradial membranes; fin spines silvery. Pectoral-fin axilla and base bright yellow. Pelvic fins white; large axillary scales on pelvic and anal fins silvery-white. Caudal fin hyaline. Snout with melanophore concentration dorsal to upper lip (Fig. 2A, B, E). In preservative: Body pale to yellowish; snout blackish due to concentrated melanophores; vertical bars on dorsal flank gray to indistinct; yellow patches on flank disappeared.

##### Distribution.

*Aurigequula fasciata* is widely distributed across the Indo-West Pacific region (Chakrabarty and Sparks 2022), with records spanning from Indonesia (White et al. 2013) and southern Indonesia to northwestern Australia (Gloerfelt-Tarp and Kailola 2022), as well as Pakistan (Psomadakis et al. 2015) and Myanmar (Psomadakis et al. 2019). In China, the species occurs in Hainan Island, the Beibu Gulf, the Nansha Islands, and the South China Sea (Zheng 1962), with rare occurrences reported from Taiwan (Chakrabarty et al. 2010; Shen and Wu 2012).

##### Remarks.

*A. fasciata* is seldom encountered in scientific surveys. This study confirms its presence in Hainan Island through specimens collected from fish markets in Lingshui and Sanya. While the species was previously recorded from Hainan and Taiwan (Zheng 1962; Chakrabarty et al. 2010), no further reliable reports have emerged in recent decades. The current population status and distribution of this species in southern Chinese coastal waters remain poorly understood and require further investigation.

### Mitochondrial genomic structure and base composition

**Figure 3.**
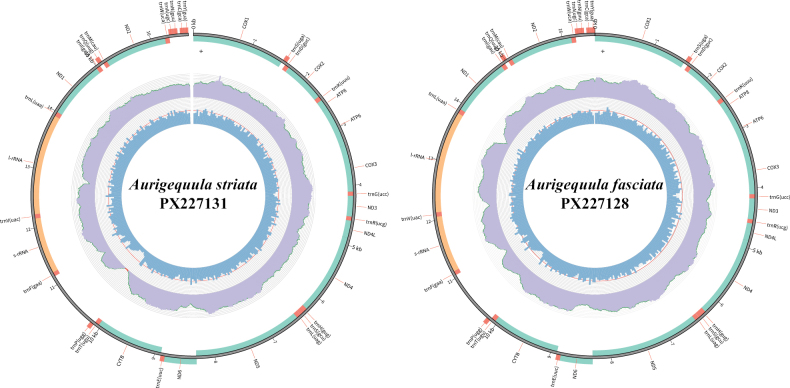
The complete mitogenome of *A. striata* and *A. fasciata*. The middle circles and innermost represent depth distribution and GC content, respectively. The outermost circle shows gene arrangements, with green for PCGs fragments, orange for rRNAs and red for tRNAs.

The complete mitogenomes of *A. striata* and *A. fasciata* were circular molecules of 16,629 bp (GenBank accession no. PX227131) and 16,537 bp (GenBank accession no. PX227128), respectively (Fig. 3, Suppl. material 1: table SS1), which is consistent with other known leiognathid species. Both mitogenomes contained the typical 37 mitochondrial genes (13 protein-coding genes, 2 rRNA genes, and 22 tRNA genes) and a major non-coding control region (Fig. 3, Suppl. material 1: table S3). With the exception of the *ND6* gene and eight tRNA genes (*trnS2*, *trnE*, *trnP*, *trnQ*, *trnA*, *trnN*, *trnC*, *trnY*), which were encoded on the light strand, all other genes were located on the heavy strand. A total of six overlapping regions (1–10 bp) were identified in the *A. striata* mitogenome, with the longest overlap (10 bp) located between *ATP8* and *ATP6*; *A. fasciata* contained five overlaps (1–10 bp), also with the longest (10 bp) between *ATP8* and *ATP6*. Additionally, ten intergenic spacers (1–40 bp) were found in *A. striata*, with the longest gap (40 bp) between *trnN* and *trnC*, whereas *A. fasciata* had nine spacers (1–39 bp), the longest of which (39 bp) was also located between *trnN* and *trnC* (Suppl. material 1: table S3).

The complete mitogenomes of both *A. striata* and *A. fasciata* exhibited a distinct AT bias, consistent with patterns observed across Leiognathidae. The overall base composition of *A. striata* was 54.7% AT (A = 30.7%, T = 24.0%, G = 14.6%, C = 30.6%), with an AT-skew of 0.123 and a GC-skew of –0.354. Similarly, *A. fasciata* had a total AT content of 54.6% (A = 30.6%, T = 24.0%, G = 15.3%, C = 30.2%), an AT-skew of 0.121, and a GC-skew of –0.329 (Suppl. material 1: tables S1, S4). In both species, the second codon position showed the highest AT content (58.8%), a characteristic feature of animal mitochondrial genomes (Zhang and Hewitt 1997; Satoh et al. 2016), while the first codon position had the lowest AT content (*A. striata* 48.3%; *A. fasciata* 48.7%) (Suppl. material 1: table S4).

### Mitochondrial protein-coding genes and codon usage

The protein-coding genes (PCGs) of both *A. striata* and *A. fasciata* spanned 11,418 bp, ranging from 177 bp (*ATP8*) to 1,830 bp (*ND5*). In both species, twelve PCGs started with the canonical ATG codon, while *COX1* used GTG as its start codon. Six PCGs possessed complete stop codons, and the remaining seven ended with incomplete termination codons T or TA—specifically, *COX2*, *ND3*, *ND4*, and *Cytb* ended with T, and *ATP6*, *COX3*, and *ND2* ended with TA (Suppl. material 1: table S3). Such incomplete stop codons are common in vertebrate mitochondrial genomes and are presumed to be completed via post-transcriptional polyadenylation (Ojala et al. 1981). The AT-skew and GC-skew values of the PCGs were 0.042 and –0.387 for *A. striata*, and 0.027 and –0.356 for *A. fasciata*, indicating a higher abundance of A and C in both species (Suppl. material 1: table S4).

**Figure 4.**
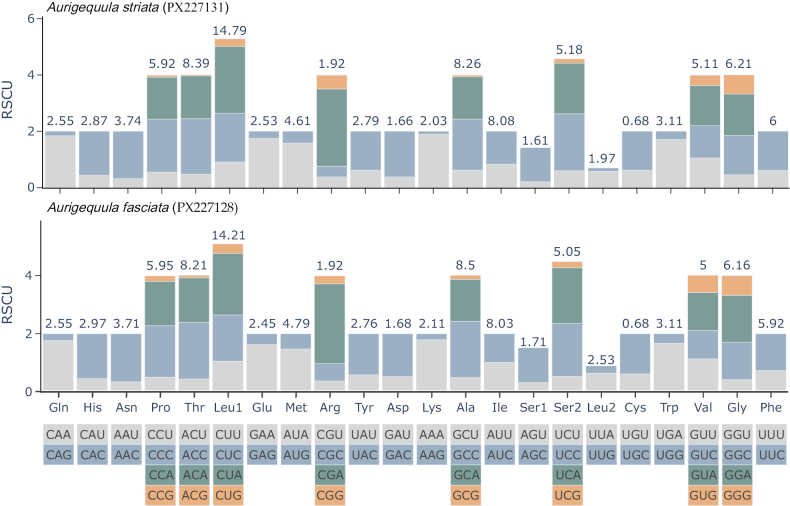
Relative synonymous codon usage of all PCGs in the mitogenome of *A. striata* and *A. fasciata*.

The amino acid usage and relative synonymous codon usage (RSCU) values in the PCGs of *A. striata* and *A. fasciata* are summarized in Suppl. material 1: table S5, Fig. 4. Both mitogenomes encoded a total of 3,806 amino acids, with leucine (14.77% in *A. striata*, 14.19% in *A. fasciata*) being the most abundant amino acid and cysteine (0.68% in both species) the least abundant. The six most frequently used codons in *A. striata* were CUA(Leu), CUC(Leu), AUC(Ile), UUC(Phe), ACC(Thr), and GCC(Ala), while those in *A. fasciata* were CUA(Leu), CUC(Leu), GCC(Ala), AUU(Ile), ACC(Thr), and AUC(Ile).

### Selection pressure analysis

**Figure 5.**
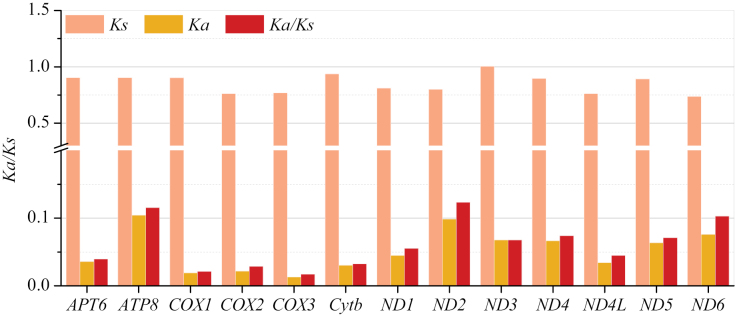
The Ka, Ks, and Ka/Ks values for each PCG from 8 Leiognathidae species mitogenomes.

In order to investigate the selective pressure on 13 PGCs of eight Leiognathidae species, we calculated the non-synonymous substitutions rate (Ka) to the synonymous substitutions rate (Ks) ratio (Ka/Ks). The Ka/Ks ratios of all PCGs were far lower than one (Fig. 5), indicating that all of the PCGs were evolving under strong purifying selection in these species (Yang and Bielawski 2000). The *ND2* gene exhibited the highest ratio (Ka/Ks = 0.1225) of all the PCGs, whereas the *COX3* gene had the lowest ratio (Ka/Ks = 0.0170).

### Mitochondrial transfer RNA and ribosomal RNA genes

The mitogenomes of both *A. striata* and *A. fasciata* contained 22 tRNA genes, ranging from 67 bp (*trnC* and *trnS1*) to 75 bp (*trnL2*) in length. These accounted for 9.33% (1,551 bp) and 9.38% (1,551 bp) of the entire mitogenomes of *A. striata* and *A. fasciata*, respectively (Suppl. material 1: table S3). In both species, 14 tRNAs were encoded on the heavy strand and eight on the light strand (Suppl. material 1: table S3), a distribution consistent with other Leiognathidae species (Shi et al. 2018; Sui et al. 2019). All tRNA genes were predicted to form typical cloverleaf secondary structures except for *trnS1*, which lacked the dihydrouridine (DHU) arm (Suppl. material 1: figs S2, S3). The overall A+T content of the tRNAs was 57.6% in both species, with a positive AT-skew (*A. striata*: 0.030; *A. fasciata*: 0.025) and GC-skew (*A. striata*: 0.064; *A. fasciata*: 0.075) (Suppl. material 1: table S4).

The 12S and 16S rRNA genes in *A. striata* and *A. fasciata* were located between *trnF* and *trnL(uaa)*, separated by *trnV(uac)*, a genomic arrangement consistent with that of other vertebrates (Fig. 3, Suppl. material 1: table S3). In *A. striata*, the 12S and 16S rRNAs were 949 bp and 1,703 bp in length, respectively, with an overall A+T content of 53.0% and G+C content of 47.0%. The AT-skew and GC-skew values were 0.303 and –0.170, respectively. In *A. fasciata*, the corresponding rRNA genes measured 950 bp (12S) and 1,699 bp (16S), with an A+T content of 52.6%, G+C content of 47.3%, AT-skew of 0.322, and GC-skew of –0.167 (Suppl. material 1: table S4). These values indicate a pronounced bias toward A and C nucleotides in the rRNA genes of both species.

### Phylogenetic analysis

**Figure 6.**
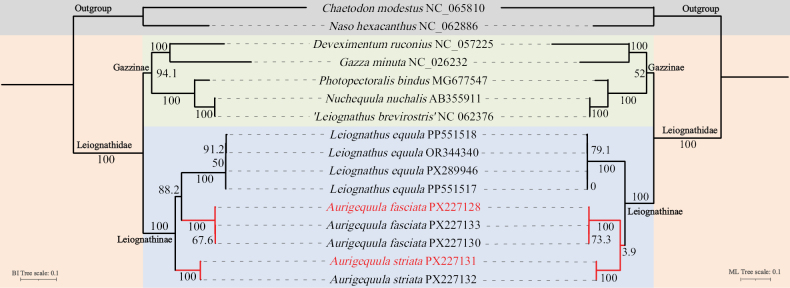
Phylogenetic tree of Leiognathidae constructed based on the amino acid sequences of 13 protein-coding genes analyzed by Bayesian inference (BI) and maximum likelihood (ML), and their groupings. Numbers above branches indicate ML bootstrap and Bayesian posterior probabilities, respectively; “*” indicates absent from maximum clade credibility tree.

The mitochondrial phylogenomic (Fig. 6) results presented herein further extend the findings of Chen et al. (2024), corroborating a distinct phylogenetic split between the subfamilies Gazzinae (comprising *Nuchequula*, *Gazza*, and *Photopectoralis*) and Leiognathinae (comprising *Aurigequula* and *Leiognathus*), each recovered as a well-supported monophyletic clade. Within Gazzinae, *Nuchequula* (which likely includes *Leiognathus brevirostris*NC_062376; see Chen et al. 2024) and *Photopectoralis* were resolved as sister genera. Similarly, *Deveximentum* (represented by *Deveximentum ruconius*, formerly *Leiognathus ruconius*) and *Gazza* formed a sister-group relationship. These relationships collectively support the monophyly of Gazzinae. In Leiognathinae, however, conflicting topologies were observed between analytical methods: the ML analysis recovered *Aurigequula* as monophyletic, though with low support (3.9/50) for its relationship to *Leiognathus*, while the BI analysis suggested paraphyly of *Aurigequula*, with *A. fasciata* forming a clade with *L. equula* to the exclusion of *A. striata*. Despite these conflicting patterns at the generic level, all examined taxa of Leiognathinae together constituted a monophyletic assemblage in both analyses.

## Discussion

We document a specimen of *A. striata* (ECSFRI 22485) with a total length of 18.8 cm (Fig. 3, Table 1), representing the second-largest individual recorded for this species, following the maximum known size of 21 cm (James and Badrudeen 1990). The original description reports eleven distinct yellowish-orange vertical bands in fresh *A. striata* (James and Badrudeen 1990), while our market-obtained specimens showed 12–15 grayish stripes. This discrepancy is consistent with the note in the original description that “bands become gray on preservation” (James and Badrudeen 1990), and likely reflects post-mortem fading of the original golden pigmentation during storage. The resulting grayish stripes bear a superficial resemblance to those of *L. equula*, indicating that coloration is an unreliable diagnostic character in non-fresh specimens.

Morphometric inconsistencies were also observed in the second dorsal-fin spine. Although the original description states that it extends to the origin of 5–11 dorsal fin rays (James and Badrudeen 1990), the spine is damaged in both our specimens, the spine is either clearly broken (ECSFRI 22484) or shows signs of damage (ECSFRI 22483). Nevertheless, in ECSFRI 22483, the spine still reaches the origin of the fifth dorsal-fin ray. Due to the uncertain condition of the spine, we provide the measured values for reference. The limited availability of reliable visual references for this species—restricted largely to the original black-and-white figure (James and Badrudeen 1990) and a single color photograph repeatedly used in publications from Sri Lanka (Chakrabarty et al. 2008) and Singapore (Chakrabarty and Sparks 2022)—highlights the importance of the detailed photographic records of two additional specimens provided herein.

The mitogenomic disparities between *Aurigequula striata* and *A. fasciata*, though subtle in overall sequence similarity, are pronounced in specific genomic architecture and compositional biases, providing compelling evidence for their genetic distinctness. The significant length variation in the non-coding control region (97 bp; Suppl. material 1: table S3), a region known for high evolutionary rates, likely reflects species-specific evolutionary trajectories. Furthermore, the substantial divergence in nucleotide composition within specific PCGs, such as the contrasting AT-skew in *ATP6* and the marked difference in AT content in *ND4L* (Suppl. material 1: table S4), indicates potential differences in mutational pressures or selective constraints acting on these genes. These findings, coupled with the distinct codon usage preference (e.g., for GCC(Ala) in *A. fasciata*; Suppl. material 1: table S5), suggest that the two species have undergone a period of independent evolution sufficient to fix characteristic molecular signatures in their mitochondrial genomes. Therefore, the concatenated evidence from both coding and non-coding regions robustly supports the validity of *A. striata* and *A. fasciata* as separate species and underscores the utility of complete mitogenomes in resolving fine-scale taxonomic relationships within the Leiognathidae.

Our mitogenomic phylogenetic analysis provides strong support for the subfamilial classification within Leiognathidae, recovering both Gazzinae and Leiognathinae as well-supported monophyletic groups in both ML and BI analyses. Considering the substantial rate heterogeneity characteristic of mitochondrial genes, we regard the Bayesian inference topology as more reliable for interpreting relationships within Leiognathinae. While our partitioned ML approach attempted to account for this variation, the Bayesian framework with more complex model parameterization (such as the GTR model; Suppl. material 1: table S2) and extensive sampling of tree space is better equipped to handle such heterogeneity. This methodological consideration is reflected in the low support value (3.9/50) at the critical node in the ML analysis, contrasted with the stable BI topology (posterior probability = 88.2).

Our study was conducted against a background of limited genomic resources for the family Leiognathidae. In particular, comprehensive mitogenomic data had been unavailable for most leiognathid genera, including the rarely encountered genus *Aurigequula*. The presentation of the first complete mitogenomes for two *Aurigequula* species here therefore represents a significant expansion of the genomic baseline for this understudied family.

It should be noted that the phylogenetic relationships inferred from our mitogenomic data differ from the framework established by Chakrabarty et al. (2011). In that multi-locus study (including nuclear genes), *Aurigequula* was recovered, together with *Leiognathus*, as a monophyletic group (subfamily Leiognathinae). In contrast, our Bayesian inference analysis based solely on mitochondrial genomes suggested the paraphyly of *Aurigequula*. This result presents a systematic dilemma when compared with clear morphological distinctions: *Aurigequula* possesses a longer second dorsal-fin spine (> 70% vs < 60% of body depth) and exhibits broader, more widely separated yellowish bars and distinct round yellowish blotches in life, unlike the narrow, closely spaced gray bars characteristic of *Leiognathus* (Chakrabarty et al. 2008; Chakrabarty and Sparks 2022). The discordance between our molecular phylogeny and these diagnostic morphological features suggests that the latter may represent homoplasy rather than synapomorphies, potentially resulting from convergent evolution or rapid morphological differentiation in recently diverged lineages. Our analysis was also constrained by the limited taxon sampling available for the rare *Aurigequula*, which may further affect topological resolution.

Thus, while the mitogenomes provided here establish an important genomic resource for future studies, a more robust phylogenetic framework for Leiognathidae—one that can reconcile molecular patterns with morphological divergence—will require the integration of multiple independent nuclear loci, following the approach demonstrated by Chakrabarty et al. (2011). Such a multi-gene dataset will be essential for critically testing hypotheses concerning generic monophyly, species boundaries, and the evolutionary history of this group.

## Conclusions

This study confirms the first record of *A. striata* in Chinese waters and provides comprehensive morphological documentation for both *A. striata* and *A. fasciata*. Mitogenomic analyses revealed structural and compositional differences supporting their status as distinct evolutionary lineages. While maximum likelihood analysis recovered *Aurigequula* as monophyletic, Bayesian inference suggested paraphyly, with *A. fasciata* clustering more closely with *L. equula* than with *A. striata*. Given the methodological strengths of Bayesian approaches in handling mitochondrial rate heterogeneity, we consider this topology more reliable. This creates a systematic dilemma, as it conflicts with key morphological diagnostic characters defining *Aurigequula*, such as the longer second dorsal-fin spine and distinct body bar patterns, implying these traits may be homoplastic.

Our research substantially enriches the scarce mitogenomic resources for Leiognathidae, particularly for the rare genus *Aurigequula*. However, the results also underscore the limitations of mitochondrial genomes alone for resolving generic relationships, contrasting with the multi-locus (including nuclear) framework established by Chakrabarty et al. (2011) that supported *Aurigequula* monophyly. Our analysis was further constrained by limited taxon sampling due to the rarity of *Aurigequula* specimens. Therefore, the genomic data presented here provide a crucial foundation, but future studies must integrate multiple independent nuclear loci, following the approach of Chakrabarty et al. (2011), to critically assess generic monophyly, species boundaries, and fully resolve the complex evolutionary history within this group.

## Supplementary Material

XML Treatment for Aurigequula striata

XML Treatment for Aurigequula fasciata

## Additional information

### Use of AI

No use of AI was reported.

### Author ORCIDs

Jia-Jie Chen https://orcid.org/0000-0001-7487-231X

Jun-Sheng Zhong https://orcid.org/0000-0002-9145-7473

Sheng Zeng https://orcid.org/0009-0001-0943-3772

De-Yuan Yang https://orcid.org/0000-0003-3735-9909

Pan Liu https://orcid.org/0009-0004-3090-4091

Xiao-Dong Wang https://orcid.org/0000-0001-9424-7896

Han-Ye Zhang https://orcid.org/0000-0002-1645-6796

Jin-Qing Ye https://orcid.org/0009-0009-6503-1477

### Data availability

All of the data that support the findings of this study are available in the main text or Supplementary Information.

## Supplementary materials

Supplementary material 1 Supplementary information Data type zip Explanation note **table SS1**. Mitochondrial sequences used in the phylogenetic analysis as shown in this study. **table S2**. Best partitioning schemes and models based on different datasets for Bayesian inference (BI) and maximum likelihood (ML) analysis. **table S3**. Information on each gene fragment of *A. striata* and *A. fasciata*. **table S4**. Base composition of the *A. striata* and *A. fasciata* mitochondrial genome. **table S5**. Codon number and RSCU of *A. striata* and *A. fasciata* mitochondrial PCGs. **figure S1**. Distribution map of collection localities for all Leiognathidae specimens in this study. **figure S2**. Secondary structure of the 22 tRNA genes of the mitochondrial genome of *A. striat*. **figure S3**. Secondary structure of the 22 tRNA genes of the mitochondrial genome of *A. fasciata*. https://binary.pensoft.net/file/1516660This dataset is made available under the Open Database License (http://opendatacommons.org/licenses/odbl/1.0/). The Open Database License (ODbL) is a license agreement intended to allow users to freely share, modify, and use this Dataset while maintaining this same freedom for others, provided that the original source and author(s) are credited.Author: Jia-Jie Chen
