# Supplementary material for: A new leiognathid record from China with complete mitogenomes and phylogenetic insights of two Aurigequula (Teleostei, Leiognathidae) species
Source: Zookeys. 2026 Jan 21;1267:31–49. doi: 10.3897/zookeys.1267.174380 (PMC12853101; doi:10.3897/zookeys.1267.174380)
Supplement: Supplementary material 1 — Supplementary information [file zookeys-1267-031_article-174380__-s001.zip › 174380_0R-1-A_Figure_S2_Secondary_structure_of_the_22_tRNA_genes_of_the_mitochondrial_genome_of_A_striat.pdf]

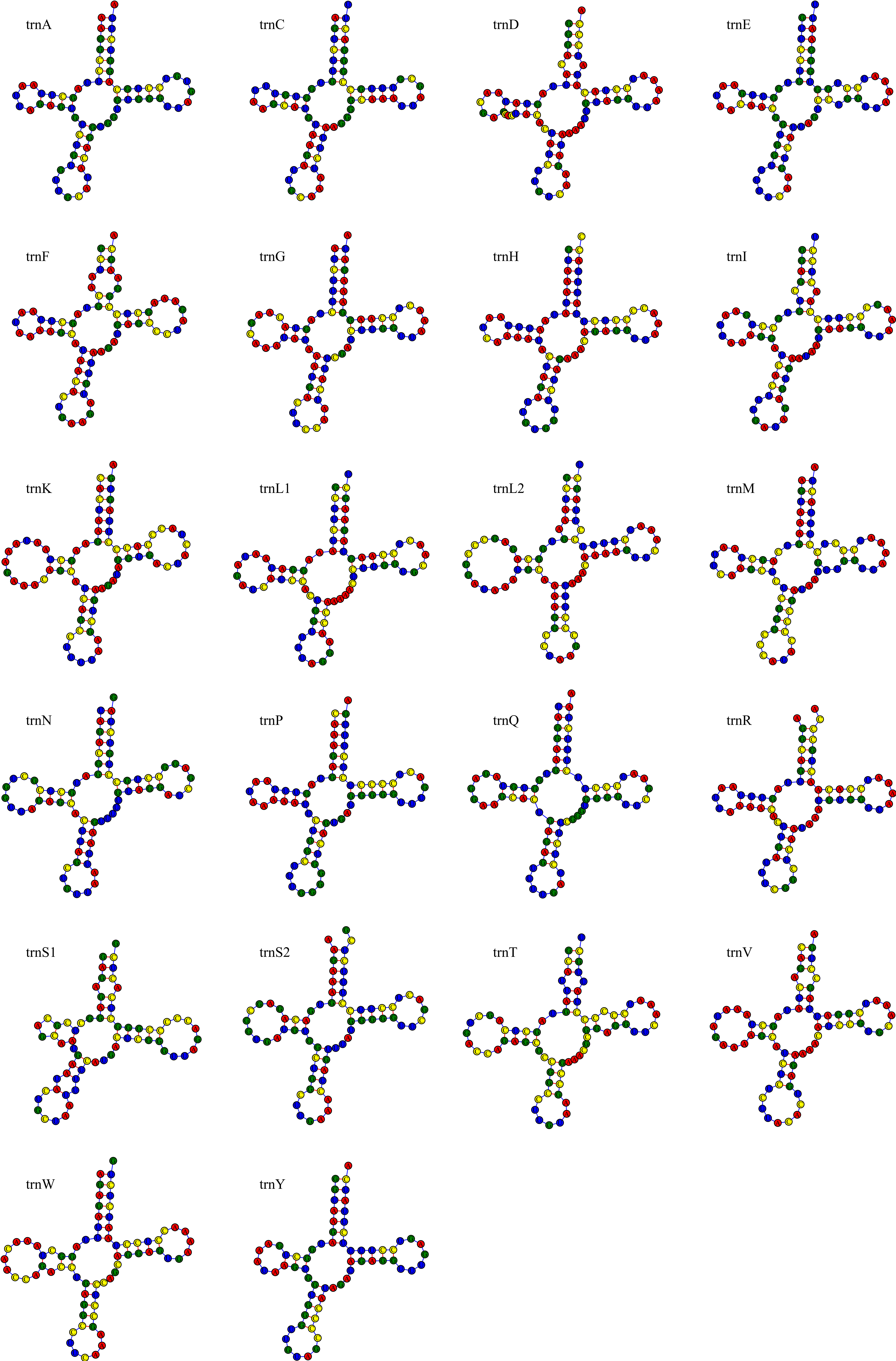
trnA
trnC
trnD
trnE
trnF
trnG
trnH
trnI
trnK
trnL1
trnL2
trnM
trnN
trnP
trnQ
trnR
trnS1
trnS2
trnT
trnV
trnW
trnY
