# Supplementary material for: A new leiognathid record from China with complete mitogenomes and phylogenetic insights of two Aurigequula (Teleostei, Leiognathidae) species
Source: Zookeys. 2026 Jan 21;1267:31–49. doi: 10.3897/zookeys.1267.174380 (PMC12853101; doi:10.3897/zookeys.1267.174380)
Supplement: Supplementary material 1 — Supplementary information [file zookeys-1267-031_article-174380__-s001.zip › 174380_1C-1-A_revised1224_Table_S1-S5_of_Aurigequula_striata.docx]

**Table S1.** Mitochondrial sequences used in the phylogenetic analysis as shown in this study.

| **Species** | **Size (bp)** | **Accession no.** | **A (%)** | **T (%)** | **C (%)** | **G (%)** | **A+T (%)** | **A+T skew** | **G+C skew** | **References** |
| --- | --- | --- | --- | --- | --- | --- | --- | --- | --- | --- |
| **Out group** |  |  |  |  |  |  |  |  |  |  |
| *Naso hexacanthus* | 16611 | [NC_062886](https://www.ncbi.nlm.nih.gov/nuccore/NC_062886) | 29.0 | 25.8 | 16.1 | 29.1 | 54.8 | 0.059 | -0.287 | (Choi et al. 2023, Chen et al. 2024) |
| *Chaetodon modestus* | 16490 | [NC_065810](https://www.ncbi.nlm.nih.gov/nuccore/NC_065810.1) | 28.0 | 28.7 | 16.7 | 26.5 | 56.7 | -0.012 | -0.227 | (Patil et al. 2022, Chen et al. 2024) |
| **Leiognathidae** |  |  |  |  |  |  |  |  |  |  |
| *Leiognathus ruconius* | 16465 | [NC_057225](https://www.ncbi.nlm.nih.gov/nuccore/NC_057225) | 31.6 | 25.2 | 14.5 | 28.7 | 56.8 | 0.112 | -0.327 | (Sui et al. 2019, Chen et al. 2024) |
| *Gazza minuta* | 16475 | [NC_026232](https://www.ncbi.nlm.nih.gov/nuccore/NC_026232) | 29.5 | 25.3 | 15.8 | 29.5 | 54.8 | 0.076 | -0.303 | (Chen et al. 2024) |
| *Photopectoralis bindus* | 16517 | [MG677547](https://www.ncbi.nlm.nih.gov/nuccore/MG677547) | 29.9 | 25.0 | 15.1 | 30.1 | 54.9 | 0.089 | -0.333 | (Shi et al. 2018, Chen et al. 2024) |
| *Nuchequula nuchalis* | 15965 | [AB355911](https://www.ncbi.nlm.nih.gov/nuccore/AB355911) | 29.8 | 25.3 | 15.2 | 29.7 | 55.1 | 0.080 | -0.322 | (Satoh et al. 2016, Chen et al. 2024) |
| *"Leiognathus brevirostris"* | 16465 | [NC_062376](https://www.ncbi.nlm.nih.gov/nuccore/NC_062376) | 29.6 | 25.5 | 15.3 | 29.6 | 55.1 | 0.074 | -0.318 | (Chen et al. 2024) |
| *Aurigequula striata* | 16629 | PX227131 | 30.7 | 24.0 | 14.6 | 30.6 | 54.7 | 0.123 | -0.354 | This study |
| *Aurigequula striata* | 16584 | PX227132 | 30.8 | 24.0 | 14.6 | 30.7 | 54.8 | 0.124 | -0.355 | This study |
| *Aurigequula fasciata* | 16489 | PX227130 | 30.5 | 23.9 | 15.3 | 30.3 | 54.4 | 0.121 | -0.330 | This study |
| *Aurigequula fasciata* | 16523 | PX227133 | 30.5 | 24.0 | 15.3 | 30.2 | 54.5 | 0.120 | -0.329 | This study |
| *Aurigequula fasciata* | 16537 | PX227128 | 30.6 | 24.0 | 15.3 | 30.2 | 54.6 | 0.121 | -0.329 | This study |
| *Leiognathus equula* | 16400 | PX289946 | 30.7 | 24.2 | 15.0 | 30.1 | 54.9 | 0.117 | -0.334 | This study |
| *Leiognathus equula* | 16398 | [OR344340](https://www.ncbi.nlm.nih.gov/nuccore/OR344340) | 30.7 | 24.2 | 15.0 | 30.1 | 54.9 | 0.117 | -0.334 | (Chen et al. 2024) |
| *Leiognathus equula* | 16399 | [PP551517](https://www.ncbi.nlm.nih.gov/nuccore/PP551517) | 30.6 | 24.3 | 15.0 | 30.1 | 54.9 | 0.117 | -0.333 | (Chen et al. 2024) |
| *Leiognathus equula* | 16395 | [PP551518](https://www.ncbi.nlm.nih.gov/nuccore/PP551518) | 30.7 | 24.2 | 15.0 | 30.1 | 54.9 | 0.117 | -0.333 | (Chen et al. 2024) |

**References**

Chen J, Wang X, Zeng S, Tian W, Yang D, Ye J, Zhong J, Jiang C (2024) Morphometric and phylogenetic analysis of a commercial fish *Leiognathus equula* (Teleostei, Leiognathidae). ZooKeys 1219: 249–270. https://doi.org/10.3897/zookeys.1219.130546

Choi H, Kim S, Choi H, Youn S (2023) The complete mitochondrial genome of sleek unicornfish, Naso hexacanthus (Acanthuridae, Perciformes). Mitochondrial DNA. Part B, Resources 8: 274–275. https://doi.org/10.1080/23802359.2022.2160666

Patil MP, Kim J-O, Lee Y-J, Seo YB, Kim J-K, Kim G-D (2022) Complete mitochondrial genome of brown-banded butterflyfish Chaetodon modestus (Chaetodontiformes, Chaetodontidae) and phylogenetic analysis. Mitochondrial DNA. Part B, Resources 7: 2012–2014. https://doi.org/10.1080/23802359.2022.2148490

Satoh TP, Miya M, Mabuchi K, Nishida M (2016) Structure and variation of the mitochondrial genome of fishes. BMC Genomics 17: 719. https://doi.org/10.1186/s12864-016-3054-y

Shi W, Wu B, Yu H (2018) The complete mitochondrial genome sequence of *Photopectoralis bindus* (Perciformes: Leiognathidae). Mitochondrial DNA Part B 3: 71–72. https://doi.org/10.1080/23802359.2017.1422404

Sui Y, Qin B, Song X, Sheng W, Zhang B (2019) Complete mitochondrial genome of the deep pugnose ponyfish *Secutor ruconius* (Perciformes: Leiognathidae) in the East China Sea. Mitochondrial DNA Part B 4: 3563–3564. https://doi.org/10.1080/23802359.2019.1670108

**Table S2** Best partitioning schemes and models based on different datasets for Bayesian inference (BI) and maximum likelihood (ML) analysis.

| **Dastaset** | **BI/ML** | **Subset partitions** | **Best model** |
| --- | --- | --- | --- |
| Migogenome | BI | P1: (ATP6_mafft_ND1_mafft_ND3_mafft) | GTR+F+I+G4 |
|  |  | P2: (ATP8_mafft_COX1_mafft_CYTB_mafft) | GTR+F+I+G4 |
|  |  | P3: (COX2_mafft_COX3_mafft_ND4L_mafft) | GTR+F+I+G4 |
|  |  | P4: (ND2_mafft_ND4_mafft_ND5_mafft) | GTR+F+I+G4 |
|  |  | P5: (ND6_mafft) | HKY+F+G4 |
|  | ML | P1: (ATP6_mafft_ND1_mafft) | TPM2u+F+I+G4 |
|  |  | P2: (ATP8_mafft) | HKY+F+G4 |
|  |  | P3: (COX1_mafft) | TIM2+F+I+I+R3 |
|  |  | P4: (COX2_mafft_COX3_mafft) | TIM2+F+I+I+R3 |
|  |  | P5: (CYTB_mafft) | TIM2+F+I+G4 |
|  |  | P6: (ND2_mafft) | TPM2u+F+I+G4 |
|  |  | P7: (ND3_mafft) | TIM2+F+I+G4 |
|  |  | P8: (ND4L_mafft) | TPM2u+F+G4 |
|  |  | P9: (ND4_mafft) | TPM2u+F+I+G4 |
|  |  | P10: (ND5_mafft) | TIM2+F+I+G4 |
|  |  | P11: (ND6_mafft) | TIM+F+G4 |

**Table S3** Information on each gene fragment of *A. striata* and *A. fasciata*

| Name | ***A. striata* (PX227131)** | | | | | | ***A. fasciata* (PX227128)** | | | | | |
| --- | --- | --- | --- | --- | --- | --- | --- | --- | --- | --- | --- | --- |
|  | **Start** | **Stop** | **Strand** | **Length** | **IGR*** | **Codons** | **Start** | **Stop** | **Strand** | **Length** | **IGR*** | **Codons** |
| *COX1* | 1 | 1551 | H | 1551 | 2 | GTG/TAA | 1 | 1551 | H | 1551 | 2 | GTG/TAA |
| *trnS2* | 1554 | 1624 | L | 71 | 3 |  | 1554 | 1624 | L | 71 | 3 |  |
| *trnD* | 1628 | 1699 | H | 72 | 7 |  | 1628 | 1699 | H | 72 | 7 |  |
| *COX2* | 1707 | 2397 | H | 691 |  | ATG/T | 1707 | 2397 | H | 691 |  | ATG/T |
| *trnK* | 2398 | 2471 | H | 74 | 1 |  | 2398 | 2471 | H | 74 | 1 |  |
| *ATP6* | 2473 | 2649 | H | 177 | -10 | ATG/TAA | 2473 | 2649 | H | 177 | -10 | ATG/TAA |
| *ATP8* | 2640 | 3322 | H | 683 |  | ATG/TA | 2640 | 3322 | H | 683 |  | ATG/TA |
| *COX3* | 3323 | 4107 | H | 785 |  | ATG/TA | 3323 | 4107 | H | 785 |  | ATG/TA |
| *trnG* | 4108 | 4177 | H | 70 |  |  | 4108 | 4177 | H | 70 |  |  |
| *ND3* | 4178 | 4526 | H | 349 |  | ATG/T | 4178 | 4526 | H | 349 |  | ATG/T |
| *trnR* | 4527 | 4594 | H | 68 |  |  | 4527 | 4594 | H | 68 | 0 |  |
| *ND4l* | 4595 | 4891 | H | 297 | -7 | ATG/TAA | 4595 | 4891 | H | 297 | -7 | ATG/TAA |
| *ND4* | 4885 | 6265 | H | 1381 |  | ATG/T | 4885 | 6265 | H | 1381 |  | ATG/T |
| *trnH* | 6266 | 6334 | H | 69 |  |  | 6266 | 6334 | H | 69 |  |  |
| *trnS1* | 6335 | 6401 | H | 67 | 4 |  | 6335 | 6401 | H | 67 | 5 |  |
| *trnL1* | 6406 | 6478 | H | 73 | 0 |  | 6407 | 6479 | H | 73 | 0 |  |
| *ND5* | 6479 | 8308 | H | 1830 | -4 | ATG/TAA | 6480 | 8309 | H | 1830 | -4 | ATG/TAA |
| *ND6* | 8305 | 8826 | L | 522 |  | ATG/TAG | 8306 | 8827 | L | 522 |  | ATG/TAG |
| *trnE* | 8827 | 8895 | L | 69 | 4 |  | 8828 | 8896 | L | 69 | 4 |  |
| *Cytb* | 8900 | 10040 | H | 1141 |  | ATG/T | 8901 | 10041 | H | 1141 |  | ATG/T |
| *trnT* | 10041 | 10114 | H | 74 | -1 |  | 10042 | 10115 | H | 74 |  |  |
| *trnP* | 10114 | 10182 | L | 69 |  |  | 10116 | 10184 | L | 69 |  |  |
| NCR | 10183 | 11139 | H | 957 |  |  | 10185 | 11044 | H | 860 |  |  |
| *trnF* | 11140 | 11207 | H | 68 |  |  | 11045 | 11112 | H | 68 |  |  |
| *12s* | 11208 | 12157 | H | 950 |  |  | 11113 | 12061 | H | 949 |  |  |
| *trnV* | 12158 | 12228 | H | 71 |  |  | 12062 | 12132 | H | 71 |  |  |
| *16s* | 12229 | 13927 | H | 1699 |  |  | 12133 | 13835 | H | 1703 |  |  |
| *trnL2* | 13928 | 14002 | H | 75 |  |  | 13836 | 13910 | H | 75 |  |  |
| *ND1* | 14003 | 14977 | H | 975 | 5 | ATG/TAA | 13911 | 14885 | H | 975 | 6 | ATG/TAA |
| *trnI* | 14983 | 15052 | H | 70 | -1 |  | 14892 | 14961 | H | 70 | -1 |  |
| *trnQ* | 15052 | 15122 | L | 71 | -1 |  | 14961 | 15031 | L | 71 | -1 |  |
| *trnM* | 15122 | 15191 | H | 70 |  |  | 15031 | 15100 | H | 70 |  |  |
| *ND2* | 15192 | 16237 | H | 1046 |  | ATG/TA | 15101 | 16146 | H | 1046 |  | ATG/TA |
| *trnW* | 16238 | 16310 | H | 73 |  |  | 16147 | 16219 | H | 73 |  |  |
| *trnA* | 16311 | 16379 | L | 69 | 1 |  | 16220 | 16288 | L | 69 | 1 |  |
| *trnN* | 16381 | 16453 | L | 73 | 40 |  | 16290 | 16362 | L | 73 | 39 |  |
| *trnC* | 16494 | 16560 | L | 67 |  |  | 16402 | 16468 | L | 67 |  |  |
| *trnY* | 16561 | 16628 | L | 68 |  |  | 16469 | 16536 | L | 68 |  |  |

* Intergenic Region: Negative numbers indicate overlapping nucleotides between adjacent genes.

**Table S4.** Base composition of the *A. striata* and *A. fasciata* mitochondrial genome.

| **Regions** | ***A. striata* (PX227131)** | | | | | | | | ***A. fasciata* (PX227128)** | | | | | | | |
| --- | --- | --- | --- | --- | --- | --- | --- | --- | --- | --- | --- | --- | --- | --- | --- | --- |
|  | **Size (bp)** | **A%** | **T%** | **C%** | **G%** | **AT(%)** | **AT skew** | **GC skew** | **Size (bp)** | **A%** | **T%** | **C%** | **G%** | **AT(%)** | **AT skew** | **GC skew** |
| Full genome | 16629 | 30.7 | 24.0 | 30.6 | 14.6 | 54.7 | 0.123 | -0.354 | 16537 | 30.6 | 24.0 | 30.2 | 15.3 | 54.6 | 0.121 | -0.329 |
| PCGs | 11418 | 27.9 | 25.7 | 32.2 | 14.2 | 53.6 | 0.042 | -0.387 | 11418 | 27.7 | 26.2 | 31.3 | 14.9 | 53.9 | 0.027 | -0.356 |
| tRNAs | 1551 | 29.7 | 27.9 | 19.9 | 22.6 | 57.6 | 0.030 | 0.064 | 1551 | 29.5 | 28.1 | 19.6 | 22.8 | 57.6 | 0.025 | 0.075 |
| rRNAs | 2649 | 34.5 | 18.5 | 27.5 | 19.5 | 53.0 | 0.303 | -0.170 | 2652 | 34.8 | 17.8 | 27.6 | 19.7 | 52.6 | 0.322 | -0.167 |
| 1st codon position | 3806 | 28.4 | 19.9 | 28.0 | 23.7 | 48.3 | 0.177 | -0.083 | 3806 | 28.5 | 20.2 | 27.6 | 23.8 | 48.7 | 0.171 | -0.074 |
| 2nd codon position | 3806 | 18.3 | 40.5 | 27.7 | 13.5 | 58.8 | -0.378 | -0.345 | 3806 | 18.4 | 40.4 | 27.7 | 13.6 | 58.8 | -0.375 | -0.342 |
| 3rd codon position | 3806 | 37.0 | 16.6 | 40.9 | 5.4 | 53.6 | 0.381 | -0.765 | 3806 | 36.1 | 18.0 | 38.6 | 7.3 | 54.1 | 0.336 | -0.684 |
| 12S rRNA | 950 | 32.6 | 17.8 | 28.7 | 20.8 | 50.4 | 0.294 | -0.159 | 949 | 33.1 | 17.8 | 28.2 | 20.9 | 50.9 | 0.300 | -0.150 |
| 16S rRNA | 1699 | 35.6 | 18.8 | 26.8 | 18.8 | 54.4 | 0.307 | -0.177 | 1703 | 35.8 | 17.9 | 27.3 | 19.1 | 53.7 | 0.334 | -0.177 |
| *ATP6* | 683 | 28.0 | 26.4 | 33.7 | 12.0 | 54.4 | 0.030 | -0.474 | 683 | 26.6 | 27.4 | 32.2 | 13.8 | 54.0 | -0.014 | -0.401 |
| *ATP8* | 177 | 31.6 | 19.8 | 40.1 | 8.5 | 51.4 | 0.231 | -0.651 | 177 | 31.1 | 22.0 | 39.0 | 7.9 | 53.1 | 0.170 | -0.663 |
| *COX1* | 1551 | 27.1 | 28.2 | 28.4 | 16.2 | 55.3 | -0.019 | -0.273 | 1551 | 26.6 | 28.2 | 28.1 | 17.2 | 54.8 | -0.029 | -0.242 |
| *COX2* | 691 | 29.5 | 24.9 | 29.7 | 15.9 | 54.4 | 0.085 | -0.302 | 691 | 28.8 | 25.0 | 29.7 | 16.5 | 53.8 | 0.070 | -0.285 |
| *COX3* | 785 | 28.2 | 24.7 | 31.6 | 15.5 | 52.9 | 0.065 | -0.341 | 785 | 27.3 | 26.4 | 30.2 | 16.2 | 53.7 | 0.017 | -0.302 |
| *Cytb* | 1141 | 27.5 | 25.5 | 33.4 | 13.6 | 53.0 | 0.038 | -0.422 | 1141 | 27.0 | 26.8 | 32.3 | 13.9 | 53.8 | 0.003 | -0.397 |
| *ND1* | 975 | 27.3 | 23.7 | 35.4 | 13.6 | 51.0 | 0.070 | -0.444 | 975 | 28.0 | 24.4 | 33.3 | 14.3 | 52.4 | 0.068 | -0.401 |
| *ND2* | 1046 | 31.4 | 21.2 | 36.9 | 10.5 | 52.6 | 0.193 | -0.556 | 1046 | 31.5 | 22.3 | 35.6 | 10.7 | 53.8 | 0.171 | -0.537 |
| *ND3* | 349 | 23.2 | 28.9 | 35.0 | 12.9 | 52.1 | -0.110 | -0.461 | 349 | 24.1 | 28.7 | 34.7 | 12.6 | 52.8 | -0.087 | -0.467 |
| *ND4* | 1381 | 28.5 | 25.0 | 34.0 | 12.5 | 53.5 | 0.066 | -0.464 | 1381 | 29.1 | 24.9 | 33.1 | 12.9 | 54.0 | 0.078 | -0.439 |
| *ND4L* | 297 | 27.6 | 24.6 | 35.4 | 12.5 | 52.2 | 0.058 | -0.479 | 297 | 25.6 | 23.9 | 35.7 | 14.8 | 49.5 | 0.034 | -0.413 |
| *ND5* | 1830 | 31.0 | 23.9 | 33.4 | 11.6 | 54.9 | 0.129 | -0.483 | 1830 | 31.2 | 24.4 | 32.5 | 12.0 | 55.6 | 0.123 | -0.461 |
| *ND6* | 522 | 12.3 | 41.6 | 12.1 | 34.1 | 53.9 | -0.544 | 0.477 | 522 | 10.9 | 41.4 | 12.1 | 35.6 | 52.3 | -0.582 | 0.494 |
| NCR | 957 | 34.4 | 35.4 | 16.9 | 13.3 | 69.8 | -0.015 | -0.121 | 860 | 32.7 | 30.9 | 20 | 16.4 | 63.6 | 0.027 | -0.099 |

**Table S5.** Codon number and RSCU of *A. striata* and *A. fasciata* mitochondrial PCGs.

| ***A. striata* (PX227131)** | | | | | | | | ***A. fasciata* (****PX227128)** | | | | | | | |
| --- | --- | --- | --- | --- | --- | --- | --- | --- | --- | --- | --- | --- | --- | --- | --- |
| **AA** | **Codon** | **Count** | **RSCU** | **AA** | **Codon** | **Count** | **RSCU** | **AA** | **Codon** | **Count** | **RSCU** | **AA** | **Codon** | **Count** | **RSCU** |
| Phe | UUU(F) | 70 | 0.61 | Tyr | UAU(Y) | 33 | 0.62 | Phe | UUU(F) | 83 | 0.74 | Ser | UCU(S) | 31 | 0.59 |
| Phe | UUC(F) | 158 | 1.39 | Tyr | UAC(Y) | 73 | 1.38 | Phe | UUC(F) | 142 | 1.26 | Ser | UCC(S) | 74 | 1.41 |
| Leu2 | UUA(L) | 62 | 0.58 | SC | UAA(*) | 5 | 3.33 | Leu | UUA(L) | 69 | 0.65 | Ser | UCA(S) | 5 | 3.33 |
| Leu2 | UUG(L) | 13 | 0.12 | SC | UAG(*) | 1 | 0.67 | Leu | UUG(L) | 27 | 0.25 | Ser | UCG(S) | 1 | 0.67 |
| Leu1 | CUU(L) | 97 | 0.91 | His | CAU(H) | 24 | 0.44 | Leu | CUU(L) | 112 | 1.06 | Pro | CCU(P) | 26 | 0.46 |
| Leu1 | CUC(L) | 184 | 1.73 | His | CAC(H) | 85 | 1.56 | Leu | CUC(L) | 169 | 1.59 | Pro | CCC(P) | 87 | 1.54 |
| Leu1 | CUA(L) | 252 | 2.37 | Gln | CAA(Q) | 89 | 1.84 | Leu | CUA(L) | 224 | 2.11 | Pro | CCA(P) | 86 | 1.77 |
| Leu1 | CUG(L) | 29 | 0.27 | Gln | CAG(Q) | 8 | 0.16 | Leu | CUG(L) | 35 | 0.33 | Pro | CCG(P) | 11 | 0.23 |
| Ile | AUU(I) | 127 | 0.83 | Asn | AAU(N) | 23 | 0.32 | Ile | AUU(I) | 155 | 1.02 | Thr | ACU(T) | 25 | 0.35 |
| Ile | AUC(I) | 180 | 1.17 | Asn | AAC(N) | 119 | 1.68 | Ile | AUC(I) | 150 | 0.98 | Thr | ACC(T) | 116 | 1.65 |
| Met | AUA(M) | 138 | 1.58 | Lys | AAA(K) | 73 | 1.9 | Met | AUA(M) | 135 | 1.48 | Thr | ACA(T) | 72 | 1.8 |
| Met | AUG(M) | 37 | 0.42 | Lys | AAG(K) | 4 | 0.1 | Met | AUG(M) | 47 | 0.52 | Thr | ACG(T) | 8 | 0.2 |
| Val | GUU(V) | 51 | 1.05 | Asp | GAU(D) | 12 | 0.38 | Val | GUU(V) | 54 | 1.14 | Ala | GCU(A) | 17 | 0.53 |
| Val | GUC(V) | 56 | 1.15 | Asp | GAC(D) | 51 | 1.62 | Val | GUC(V) | 46 | 0.97 | Ala | GCC(A) | 47 | 1.47 |
| Val | GUA(V) | 69 | 1.42 | Glu | GAA(E) | 84 | 1.75 | Val | GUA(V) | 62 | 1.31 | Ala | GCA(A) | 76 | 1.63 |
| Val | GUG(V) | 18 | 0.37 | Glu | GAG(E) | 12 | 0.25 | Val | GUG(V) | 28 | 0.59 | Ala | GCG(A) | 17 | 0.37 |
| Ser2 | UCU(S) | 26 | 0.6 | Cys | UGU(C) | 8 | 0.62 | Tyr | UAU(Y) | 23 | 0.54 | Cys | UGU(C) | 8 | 0.62 |
| Ser2 | UCC(S) | 87 | 2.02 | Cys | UGC(C) | 18 | 1.38 | Tyr | UAC(Y) | 78 | 1.82 | Cys | UGC(C) | 18 | 1.38 |
| Ser2 | UCA(S) | 77 | 1.79 | Trp | UGA(W) | 101 | 1.71 | SC* | UAA(*) | 82 | 1.91 | Trp | UGA(W) | 99 | 1.68 |
| Ser2 | UCG(S) | 7 | 0.16 | Trp | UGG(W) | 17 | 0.29 | SC* | UAG(*) | 9 | 0.21 | Trp | UGG(W) | 19 | 0.32 |
| Pro | CCU(P) | 31 | 0.55 | Arg | CGU(R) | 7 | 0.38 | His | CAU(H) | 29 | 0.51 | Arg | CGU(R) | 7 | 0.38 |
| Pro | CCC(P) | 106 | 1.88 | Arg | CGC(R) | 7 | 0.38 | His | CAC(H) | 100 | 1.77 | Arg | CGC(R) | 11 | 0.6 |
| Pro | CCA(P) | 84 | 1.49 | Arg | CGA(R) | 50 | 2.74 | Gln | CAA(Q) | 86 | 1.52 | Arg | CGA(R) | 50 | 2.74 |
| Pro | CCG(P) | 4 | 0.07 | Arg | CGG(R) | 9 | 0.49 | Gln | CAG(Q) | 11 | 0.19 | Arg | CGG(R) | 5 | 0.27 |
| Thr | ACU(T) | 38 | 0.48 | Ser1 | AGU(S) | 9 | 0.21 | Asn | AAU(N) | 35 | 0.45 | Ser | AGU(S) | 14 | 0.33 |
| Thr | ACC(T) | 157 | 1.97 | Ser1 | AGC(S) | 52 | 1.21 | Asn | AAC(N) | 151 | 1.94 | Ser | AGC(S) | 51 | 1.19 |
| Thr | ACA(T) | 121 | 1.52 | SC | AGA(*) | 0 | 0 | Lys | AAA(K) | 119 | 1.53 | SC* | AGA(S) | 0 | 0 |
| Thr | ACG(T) | 3 | 0.04 | SC | AGG(*) | 0 | 0 | Lys | AAG(K) | 7 | 0.09 | SC* | AGG(S) | 0 | 0 |
| Ala | GCU(A) | 49 | 0.62 | Gly | GGU(G) | 27 | 0.46 | Asp | GAU(D) | 40 | 0.5 | Gly | GGU(G) | 25 | 0.43 |
| Ala | GCC(A) | 142 | 1.81 | Gly | GGC(G) | 82 | 1.39 | Asp | GAC(D) | 156 | 1.93 | Gly | GGC(G) | 75 | 1.28 |
| Ala | GCA(A) | 118 | 1.5 | Gly | GGA(G) | 87 | 1.47 | Glu | GAA(E) | 116 | 1.44 | Gly | GGA(G) | 94 | 1.61 |
| Ala | GCG(A) | 5 | 0.06 | Gly | GGG(G) | 40 | 0.68 | Glu | GAG(E) | 11 | 0.14 | Gly | GGG(G) | 40 | 0.68 |

*Stop codon
