# Supplementary figures and images for: A new leiognathid record from China with complete mitogenomes and phylogenetic insights of two Aurigequula (Teleostei, Leiognathidae) species
Source: Zookeys. 2026 Jan 21;1267:31–49. doi: 10.3897/zookeys.1267.174380 (PMC12853101; doi:10.3897/zookeys.1267.174380)

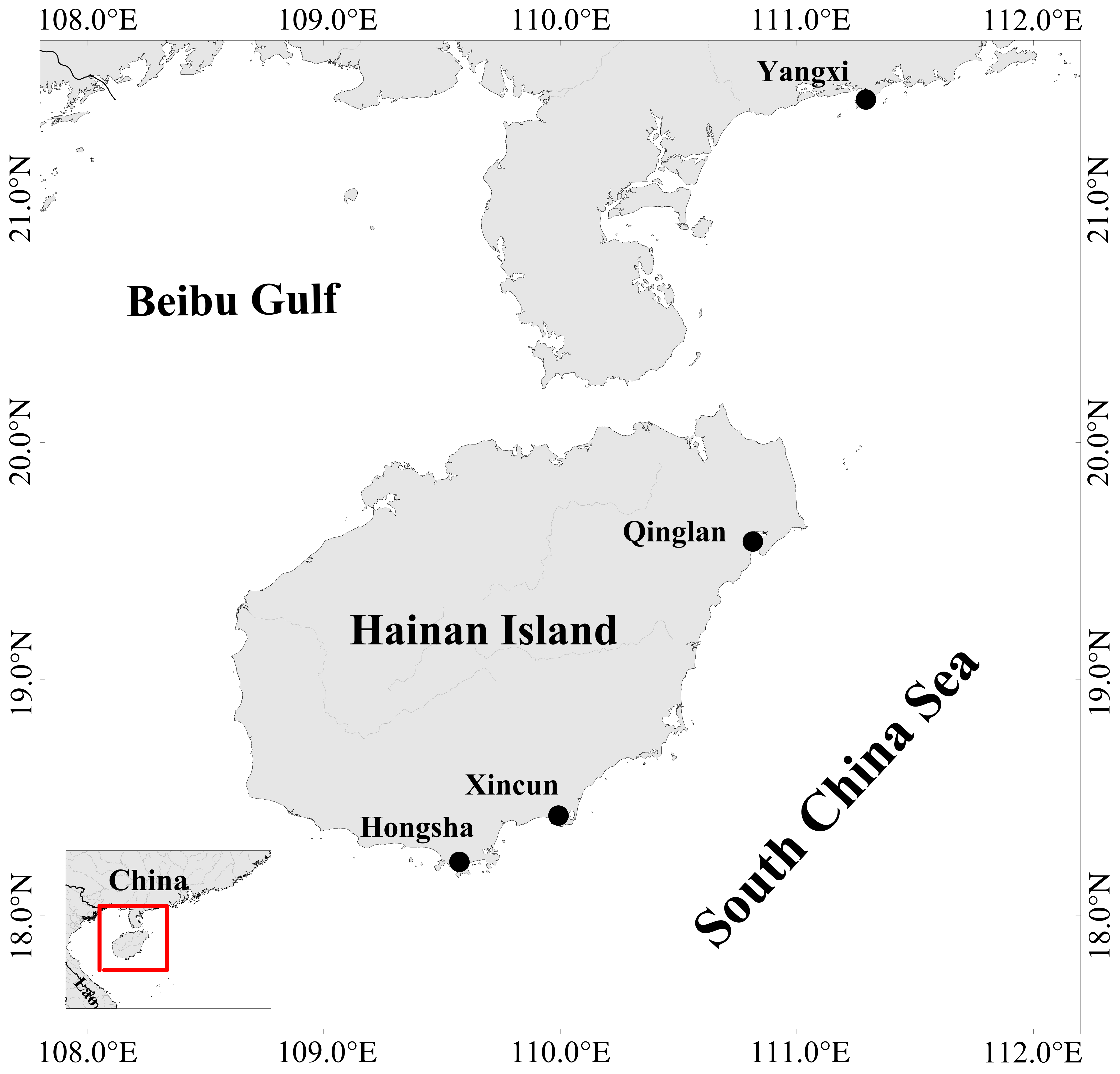

Supplement: Supplementary material 1 — Supplementary information [file zookeys-1267-031_article-174380__-s001.zip › 174380_1C-1-A_revised_add_Figure_S1_location.pdf]
